# Subtype‐specific differences in transmission cluster dynamics of HIV‐1 B and CRF01_AE in New South Wales, Australia

**DOI:** 10.1002/jia2.25655

**Published:** 2021-01-20

**Authors:** Francesca Di Giallonardo, Angie N Pinto, Phillip Keen, Ansari Shaik, Alex Carrera, Hanan Salem, Christine Selvey, Steven J Nigro, Neil Fraser, Karen Price, Joanne Holden, Frederick J Lee, Dominic E Dwyer, Benjamin R Bavinton, Jemma L Geoghegan, Andrew E Grulich, Anthony D Kelleher, the NSW HIV Prevention Partnership Project

**Affiliations:** ^1^ The Kirby Institute The University of New South Wales Sydney NSW Australia; ^2^ Royal Prince Alfred Hospital Sydney NSW Australia; ^3^ NSW HIV Reference Laboratory Sydney NSW Australia; ^4^ New South Wales Health Pathology‐RPA Royal Prince Alfred Hospital Camperdown NSW Australia; ^5^ Health Protection NSW Sydney NSW Australia; ^6^ Positive Life New South Wales Sydney NSW Australia; ^7^ ACON Sydney NSW Australia; ^8^ NSW Ministry of Health Sydney NSW Australia; ^9^ Sydney Medical School University of Sydney Sydney NSW Australia; ^10^ New South Wales Health Pathology‐ICPMR Westmead Hospital Westmead NSW Australia; ^11^ Department of Microbiology and Immunology University of Otago Dunedin New Zealand; ^12^ Institute of Environmental Science and Research Wellington New Zealand

**Keywords:** HIV1, transmission cluster, subtype B and CRF01_AE, demographic differences, early infections, public health

## Abstract

**Introduction:**

The human immunodeficiency virus 1 (HIV‐1) pandemic is characterized by numerous distinct sub‐epidemics (clusters) that continually fuel local transmission. The aims of this study were to identify active growing clusters, to understand which factors most influence the transmission dynamics, how these vary between different subtypes and how this information might contribute to effective public health responses.

**Methods:**

We used HIV‐1 genomic sequence data linked to demographic factors that accounted for approximately 70% of all new HIV‐1 notifications in New South Wales (NSW). We assessed differences in transmission cluster dynamics between subtype B and circulating recombinant form 01_AE (CRF01_AE). Separate phylogenetic trees were estimated using 2919 subtype B and 473 CRF01_AE sequences sampled between 2004 and 2018 in combination with global sequence data and NSW‐specific clades were classified as clusters, pairs or singletons. Significant differences in demographics between subtypes were assessed with Chi‐Square statistics.

**Results:**

We identified 104 subtype B and 11 CRF01_AE growing clusters containing a maximum of 29 and 11 sequences for subtype B and CRF01_AE respectively. We observed a > 2‐fold increase in the number of NSW‐specific CRF01_AE clades over time. Subtype B clusters were associated with individuals reporting men who have sex with men (MSM) as their transmission risk factor, being born in Australia, and being diagnosed during the early stage of infection (*p* < 0.01). CRF01_AE infections clusters were associated with infections among individuals diagnosed during the early stage of infection (*p* < 0.05) and CRF01_AE singletons were more likely to be from infections among individuals reporting heterosexual transmission (*p* < 0.05). We found six subtype B clusters with an above‐average growth rate (>1.5 sequences / 6‐months) and which consisted of a majority of infections among MSM. We also found four active growing CRF01_AE clusters containing only infections among MSM. Finally, we found 47 subtype B and seven CRF01_AE clusters that contained a large gap in time (>1 year) between infections and may be indicative of intermediate transmissions via undiagnosed individuals.

**Conclusions:**

The large number of active and growing clusters among MSM are the driving force of the ongoing epidemic in NSW for subtype B and CRF01_AE.

## 1 Introduction

Australia is at the forefront of successful control of human immunodeficiency virus type 1 (HIV‐1) transmission due to rapid and comprehensive public health responses from the beginning of the epidemic in the 1980s. Australia has recently experienced its first major decline in new HIV‐1 infections in ten years that is attributed to the successful rapid rollout of pre‐exposure prophylaxis (PrEP) among men who have sex with men (MSM) [1, 2]. New South Wales (NSW) the most populated state in Australia, accounts for approximately one‐third of all HIV‐1 notifications in the country; of the 963 new HIV‐1 notifications reported in 2017 in Australia, 349 were from NSW [1, 3]. NSW met the UNAIDS 90‐90‐90 targets in 2016 [4] and the number of new HIV‐1 notification decreased by 13% from 318 in 2016 to 277 in 2018. However, a slight increase in new HIV‐1 notification was reported for 2019 (n = 282) [3]. Thus, current enhanced prevention strategies alone are not sufficient to virtually eliminate HIV‐1 transmission.

HIV‐1 transmission is often characterized by the presence of numerous transmission clusters that are likely to play a key role in sustaining the epidemic and thus should be prioritized for HIV prevention [5, 6]. Phylogenetic analysis provides a powerful tool to investigate HIV‐1 transmission dynamics and has been used to determine epidemiological parameters, such as the transmission rate (i.e. basic reproductive number, R_0_) [7] and to what extent HIV‐1 transmission clusters are self‐sustained [5]. Molecular phylogeny can identify transmission clusters that would otherwise be missed by patient demographic data alone [8], particularly for new HIV‐1 notifications that are made during the chronic or advanced stages of infection [9, 10]. The fine detail of transmission dynamics of HIV‐1 in Australia is not well understood, as current surveillance reports rely on basic demographic data alone and do not include molecular epidemiological data [1]. Specifically, it is not known if the subtype B epidemic is characterized by many small transmission clusters, as often observed in other countries [11], or fewer but larger sub‐epidemics that are more common in regions with less population migration [12]. In addition, it remains unclear to what extent demographic factors such as transmission risk factor or stage of infection at diagnosis differ between clusters and subtypes. These are key factors that, if known, could improve Australia’s public health response [13].

The aim of this study was to understand changes in cluster dynamics over time in NSW. We performed phylogenetic analyses to identify sequence clusters that represent sub‐epidemics in the two most common HIV‐1 subtypes B and CRF01_AE [14] and followed the transmission dynamics in these clusters across a five‐year time period.

## 2 Methods

### 2.1 Ethics

Ethical approval was obtained by the NSW Population and Health Services Research Ethics Committee and the ACON Research Ethics Review Committee (RERC) [AU RED Reference: HREC/15/CIHS/38, Cancer Institute NSW reference number: 2015/08/605]. The HIV/AIDS Legal Centre was consulted for legal advice on data anonymity. Both, sequence and demographic data are routinely collected and are irreversibly de‐identified for public health surveillance. A waiver for consent of the individual to use their health information was granted by the Ethics Committee.

### 2.2 Sequence data and phylogeny

The NSW HIV‐1 database contains all *protease*, *reverse transcriptase* and *integrase* sequences sampled in NSW between 2004 and 2018. The HIV‐1 subtype for these sequences was determined using the Stanford HIV subtyping tool [15, 16] and confirmed by phylogeny, as described previously [14]. Only the first available sequence for each individual covering the *protease* and *reverse transcriptase* and that were classified as subtype B and CRF01_AE were included in the analysis. The final NSW data set consisted of 2919 subtype B and 473 CRF01_AE sequences. Global sequences (n = 13 194 subtype B; n = 4091 CRF01_AE) were selected via BLASTN search to identify those global sequences that are most similar to the sequences used in this study, and thus, represent background data to the NSW data [5, 17, 18]. A subtype C sequence (accession number AY162223) and a subtype B sequence (accession number NC_001802) was added as an outgroup to the subtype B and CRF01_AE data set respectively. Sequences were aligned in MAFFT [19] implementing the L‐INS‐I algorithm. Alignments were visually inspected in Geneious 11.1.3 (https://www.geneious.com) and codons associated with drug resistance mutations were removed (according to reference sequence HXB2; accession number K03455.1) [20]. Phylogenetic trees were estimated using FastTreeMP v2.1.10 [21] (Figure S1).

### 2.3 Data subsets

A baseline data set was established using NSW sequences from 2004 to 2012 plus global sequences derived from the Los Alamos National Lab (LANL) database (http://www.hiv.lanl.gov/). This global sequence data set was used as background to identify and separate out local transmission clusters within NSW. Only clusters not including global sequences were counted as true local clusters (see below). This approach has been widely established as a robust alternative to other cluster identification methods [5, 17]. In brief, each HIV‐1 sequence from NSW used in this study was compared against the LANL database via BLASTN and the 50 best matches were extracted. Thus the baseline data set contains NSW sequences from infections sampled between 2004 and 2012 plus global sequences (no time constraints for the latter). This baseline data set represents the basis for cluster growth estimates. The 2013 cut‐off was used as it represents the first full year after a new five‐year NSW HIV Strategy was released. This strategy outlines changes in public health approaches to HIV prevention for the years 2012 to 2016 [22]. NSW sequences sampled from 2013 were added to the baseline data set in six‐month intervals forming 12 data subsets for subtype B and 12 data subsets for CRF01_AE (Figure S1). Sequence phylogeny was estimated separately for each of the 12 subtype B and 12 CRF01_AE data subsets. Sequence phylogeny for the complete data set, that is global data plus NSW sequences 2004 to 2018 are shown in Figure S2.

### 2.4 Characterization of NSW‐specific clusters

An internal *R* script was used to extract clades within the phylogeny that only contained sequences from NSW, that is were monophyletic [14, 17]. NSW‐specific sequences that fell into neither category were defined as singletons (Figure S1). Clusters and pairs identified at baseline were followed across time between 2013 and 2018. If a sequence from a new data subset fell into one of these clades they were classified as “growing.” Thus, clusters were then separated into those that grew in size over time and those that did not (Figure S1). Some clusters displayed phylogenetic instability through time, that is their topology in the trees was unstable due to poor node support, and thus were not included in the analysis of cluster growth.

### 2.5 Demographic factors

Metadata derived from the notifications database included: (i) sex: male, female; (ii) self‐reported region of acquisition: Australia, not Australia; (iii) region of birth: Australian, non‐Australian, and the latter further subdivided into Asian, European and other; (iv) transmission risk factor: MSM (men who have sex with men), heterosexual, PWID (people who inject drugs), other and (v) stage of infection at diagnosis. Infection stage categories were adapted from the NSW Ministry of Health definitions [23]: early = evidence of an HIV‐1 infection acquired within 12 months of diagnosis or CD4 T‐cell count > 500 cells/mm^3^, CD4 < 500 (cell count 350 to 499 cells/mm^3^), CD4 < 350 (cell count 200 to 349 cells/mm^3^) and advanced = CD4 count <200 cells/mm^3^ or AIDS defining illness in absence of early diagnosis. Time intervals from infection to CD4 T‐cell counts at diagnosis were derived from the results of the CASCADE study [24]: early = <1 year, CD4 < 500 = 1 year, CD4 < 350 = 4 years, and CD4 < 200 = 8 years. Chi‐square tests of independence were performed in *R* (version 3.6.2) using the *gplots* and *corrplots* packages [25, 26, 27].

## 3 Results

### 3.1 Subtype‐specific differences in cluster dynamics over time

**Figure 1:**
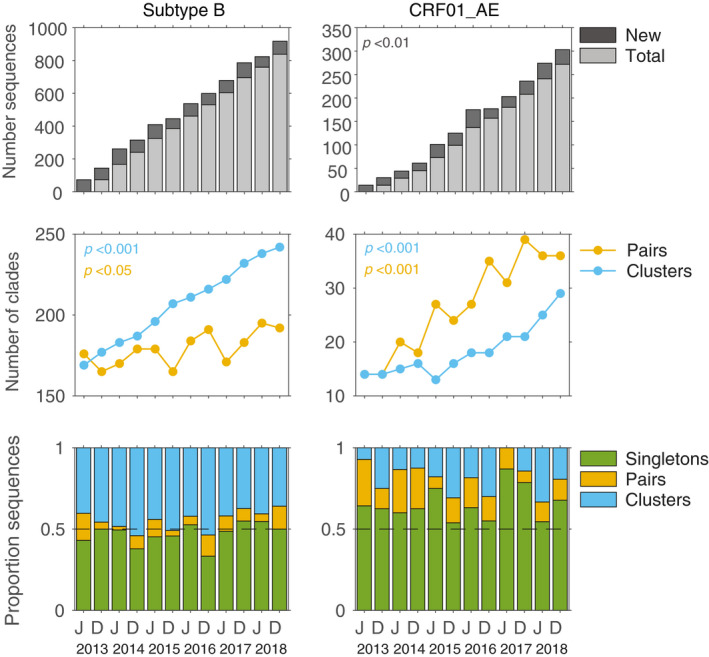
Number of clusters for subtype B and CRF01_AE between 2013 and 2018. Top Panel The total number of sequences (light grey) and new sequences added (dark grey) accumulating over time. Middle Panel: The total number of pairs (yellow) and clusters (light blue) identified across all data subsets. Note the different y‐axis scales for subtype B and CRF01_AE. Lower Panel: Proportion of sequences from newly notified infections belonging to a cluster (light blue), a sequence pair (yellow), or being a singleton (green) across the different data subsets. D, interval ending in December; J, interval ending in June.

The sequences with linked demographics used in this study cover approximately 70% of newly notified infections and represent even greater proportions for recent years (87% and 89% for 2017 and 2018 respectively). The number of sequences from new notifications used in the data subsets did not increase over time for subtype B (*p* = 0.957) but did for CRF01_AE (*p* < 0.01), increasing from 14 sequences in June 2013 to 31 in December 2018 (Figure 1). Nevertheless, the cumulative number of subtype B pairs increased slightly over time from 176 to 192 between June 2013 and December 2018 (*p* < 0.05), and the number of B clusters increased substantially from 169 in the interval ending June 2013 to 242 clusters in the interval ending December 2018 (*p* < 0.001). For every six months interval, more B clusters than sequence pairs were found, except for the first six months of 2013 when there were 176 subtype B sequence pairs and 169 clusters. Also, sequence pairs represented 51% of all NSW‐specific subtype B nodes in the interval to June 2013 and 44% in the interval ending December 2018. The opposite was observed for CRF01_AE. More sequence pairs were identified than clusters for each interval from June 2014 to December 2018 (Figure 1). Within the time period June 2013 to December 2018, 14 sequence pairs and 14 clusters were identified. Both, the number of CRF01_AE pairs and clusters increased by >2‐fold in these five years (*p* < 0.001) from 14 to 36 and 14 to 29 respectively (Figure 1). Overall, the majority of subtype B infections were associated with a clade. The lowest proportion of subtype B singletons (33%) was identified in the interval to December 2016, whereas the highest (55%) was in the interval to December 2017 (Figure 1B). In stark contrast, the majority of CRF01_AE infections were singletons (average 65%). The highest proportion of CRF01_AE singletons was observed in the interval ending June 2017 (87%) and the lowest proportion was observed in the interval ending December 2015 (54%). No difference in the proportion of singletons was found over time in either subtype.

### 3.2 Subtype‐specific differences in demographics associated with sequence clusters

**Figure 2:**
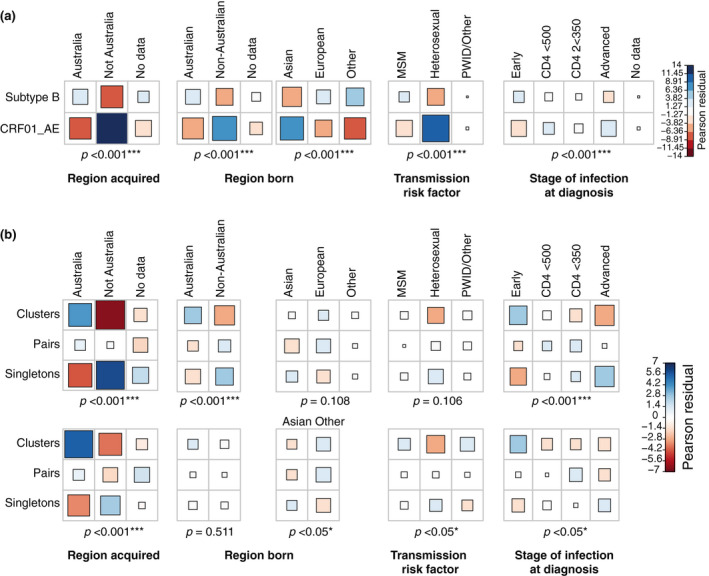
Correlations between sequence demographics and cluster association. Correlation plots shows the Chi‐square statistics for demographics. For each association (cell) the Pearson residual value is shown. A positive association is indicated in blue, a negative association is shown in red. More intense colours and larger squares equal stronger contribution to the overall Chi‐square score, and which are indicated below each correlation plot. As there were less than five sequences with the PWID transmission risk factor, this risk factor category was combined with “Other”. (**A**) Demographics compared to all subtype B and CRF01_AE, (**B**) Demographics compared to infections associated with clusters, pairs and singletons for subtype B (top panel) and CRF01_AE (bottom panel). MSM, men who have sex with men; PWID, person who inject drugs.

There were strong associations between demographic factors such as region acquired, region of birth, transmission risk factor, and stage of infection at diagnosis and subtype. Sex was not included in the statistical analysis due to a lack of demographic variation (Table 1). Infections acquired in Australia were positively associated with being of subtype B origin, whereas infections acquired elsewhere were associated with being CRF01_AE (*p* < 0.001, Figure 2A). Similarly, infections in individuals born in Australia were associated with being subtype B and infections in individuals not born in Australia were more likely to be CRF01_AE (*p* < 0.001). More specifically, infections among Asian‐born individuals were associated with being CRF01_AE, whereas infections among European‐born individuals were associated with being subtype B (*p* < 0.001). For both subtypes, the majority of infections were acquired via MSM transmission (86% subtype B, 64% CRF01_AE). However, significantly more CRF01_AE infections were acquired via heterosexual transmission (29%) compared to subtype B (8%, <0.001). In addition, CRF01_AE was more likely associated with HIV notifications identified during the advanced stage of infection (29% of CRF01_AE), compared to 19% of subtype B infections being from individuals in the advanced stage of infection (*p* < 0.001).

**Table 1:** Summary of demographic factors for subtype B and CRF01_AE infections

	Subtype B				CRF01_AE			
	All (n = 1666)	Clusters (n = 733, 44%)	Pairs (n = 146, 9%)	Singletons (n = 787, 47%)	All (n = 406)	Clusters (n = 76, 19%)	Pairs (n = 62, 15%)	Singletons (n = 268, 66%)
*Sex*								
Male	0.82 (1378)	0.83 (609)	0.84 (122)	0.81 (636)	0.72 (293)	0.86 (65)	0.274 (46)	0.68 (182)
Female	0.02 (37)	0.02 (16)	0.02 (4)	0.02 (17)	0.09 (37)	0.07 (5)	0.11 (7)	0.09 (25)
No data	0.82 (1378)	0.83 (609)	0.84 (122)	0.81 (636)	0.72 (293)	0.86 (65)	0.274 (46)	0.68 (182)
Region acquired								
Australia	0.60 (1002)	0.72 (525)	0.66 (96)	0.48 (381)	0.31 (125)	0.66 (50)	0.37 (23)	0.19 (52)
Not Australia	0.15 (243)	0.06 (41)	0.16 (23)	0.23 (179)	0.55 (222)	0.24 (18)	0.40 (25)	0.67 (179)
No data	0.25 (421)	0.23 (167)	0.18 (27)	0.29 (227)	0.14 (59)	0.11 (8)	0.23 (14)	0.14 (37)
Region born								
Australian	0.62 (1038)	0.68 (495)	0.58 (85)	0.57 (449)	0.33 (134)	0.38 (29)	0.34 (21)	0.31 (84)
Non‐Australian	0.33 (561)	0.27 (201)	0.140 (58)	0.38 (298)	0.65 (264)	0.61 (46)	0.61 (38)	0.68 (180)
Asia	0.11 (188)	0.09 (65)	0.09 (13)	0.14 (110)	0.53 (216)	0.43 (33)	0.45 (28)	0.58 (155)
Europe	0.09 (156)	0.08 (62)	0.15 (22)	0.09 (72)	—	—	—	—
Other	0.12 (213)	0.10 (74)	0.16 (23)	0.15 (116)	0.12 (48)*	0.17 (13)*	0.16 (10)*	0.09 (25)*
No data	0.05 (81)	0.05 (37)	0.02 (3)	0.05 (40)	0.02 (8)	0.01 (1)	0.05 (3)	0.01 (4)
Transmission risk factor								
MSM	0.86 (1445)	0.88 (642)	0.86 (125)	0.85 (667)	0.64 (260)	0.74 (56)	0.61 (38)	0.62 (166)
Heterosexual	0.08 (138)	0.06 (45)	0.10 (14)	0.10 (77)	0.29 (116)	0.14 (11)	0.31 (19)	0.32 (86)
PWID/Other	0.06 (97)	0.06 (46)	0.05 (7)	0.05 (43)	0.07 (30)	0.12 (9)	0.08 (5)	0.06 (16)
Stage of infection at diagnosis								
Early	0.56 (943)	0.64 (467)	0.53 (77)	0.50 (392)	0.40 (162)	0.57 (43)	0.42 (26)	0.35 (93)
CD4 < 500	0.11 (183)	0.10 (75)	0.13 (19)	0.11 (88)	0.15 (61)	0.11 (8)	0.15 (9)	0.16 (44)
CD4 < 350	0.11 (177)	0.09 (67)	0.14 (20)	0.11 (90)	0.13 (53)	0.09 (7)	0.19 (12)	0.13 (34)
Advanced	0.19 (319)	0.14 (103)	0.18 (27)	0.24 (185)	0.29 (117)	0.21 (16)	0.21 (13)	0.33 (88)
No data	0.03 (58)	0.03 (21)	0.02 (3)	0.04 (32)	0.03 (13)	0.03 (2)	0.03 (2)	0.03 (9)

As there were less than five sequences with the PWID transmission risk factor, this risk factor category was combined with “Other”. In the category “Region born" non‐Australian is further subdivided into born in Asia, Europe and others for subtype B, and born in Asia and others for CRF01_AE. MSM, men who have sex with men; PWID, person who inject drugs.

Overall, 44% and 9% of subtype B, and 19% and 15% of CRF01_AE infections were associated with clusters and pairs respectively (Table 1). Infections acquired in Australia were associated with being in clusters for both subtype B and CRF01_AE, whereas infections not acquired in Australia were associated with being singletons for both subtypes (*p* < 0.001). This latter association was stronger for subtype B than CRF01_AE (Figure 2B). For subtype B, there was a positive association for infections in non‐Australian born individuals (38%), and for infections diagnosed at the advanced stage of infection (24%) with being singletons (Figure 2; *p* < 0.001). No difference was found for infections in non‐Australian‐born individuals from different regions between being either in clusters or not (*p* = 0.108). In contrast, infections in Australian‐born, and individuals in their early stage of infection had a positive association with being part of a cluster (27% and 64% respectively; *p* < 0.001). No associations were found for any of the transmission risk factors and being in a cluster or pair, or being a singleton (*p* = 0.106).

The proportion of infections among Australian‐born and non‐Australian born individuals were not significantly different in CRF01_AE clusters, pairs, or being singletons (*p* = 0.511, Figure 2B). However, infections in Asian‐born individuals were associated with being singletons, but infections in individuals born in other regions were associated with clusters (*p* < 0.05). Infections in individuals diagnosed during the early stage of infection showed a strong positive correlation with being in a cluster, representing 57% of all infections associated with clusters (*p* = 0.05). Also, more CRF01_AE infections in individuals diagnosed at an advanced stage of infection were classified as singletons (33%) than in clusters (21%). There was a negative association for infections associated with heterosexual transmission and belonging to a cluster (*p* = 0.05), with 14% of sequences in clusters derived from infections acquired via heterosexual transmission and this risk factor contributed to 31% and 32% for pairs and singletons respectively (Table 1).

### 3.3 Large delay in notifications of new infections

**Figure 3:**
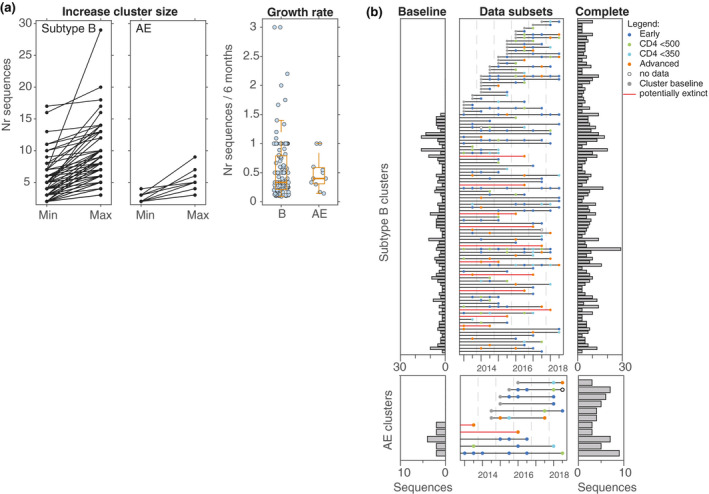
Time intervals between sampling of infections in growing clusters. (**A**) (left and middle panels) Changes in cluster size shown as the difference between the minimum number of sequences when the cluster was first identified in the phylogeny to the maximum number of sequences when the most recent sequence associated with that cluster was sampled. (right panel) Cluster growth rate was normalised to the number of sequences added per 6‐months interval. (**B**) Cluster size at baseline (including sequence data from 2004 to 2012). (middle) Each line represents a cluster and the circles represent a sequence sampled during that data subset coloured according to the stage of infection at diagnosis. Blue = early, green = CD4 T‐cell count < 500, light blue CD4 < 350, red = advanced (see Material and Methods), white = no data. Grey circles represent the starting point of clusters that appeared between 2013 and 2018. Red lines indicated clusters that only contained sequences sampled during the advanced stage of infection for sampling dates 2013 ‐ 2018 and thus are regarded as potentially extinct. (right) Final cluster size at the end of the time period investigated. Top panels show data for subtype B, bottom panels show data for CRF01_AE. D, December; J, June.

Fifty‐two subtype B and seven CRF01_AE clusters were identified in the June 13 data subset that did not grow in size in the subsequent five years. These “non‐growing” clusters were regarded as potentially extinct. In contrast, 104 subtype B and 11 CRF01_AE clusters grew in size (Figure 3A). Between 2013 and 2018, 43% of growing subtype B clusters increased by only one infection, 39% by two, three or four, and 20% by five or more infections. Overall, the largest increase in cluster size was from seven in 2013 to 29 infections in 2018 which was also the largest subtype B cluster identified. The largest increase in cluster size was also seen in the biggest CRF01_AE cluster growing from three in 2013 to nine infections in 2018. A second cluster grew by five and four (36% of growing CRF01_AE clusters) grew by only one and five (45%) by two or three infections from 2013 to 2018. Also, 39 subtype B clusters (38% of growing B clusters) started as sequence pairs, developing into clusters with up to nine infections by 2018. For CRF01_AE, seven out of the 11 growing clusters (63%) started as pairs with one growing to seven sequences by December 2018.

The cluster growth rate did not differ between the two subtypes. The median rate for cluster growth was 0.33 and 0.4 infections per six‐month interval for subtype B and CRF01_AE respectively (Figure 3A). Two CRF01_AE clusters grew at a rate of 1 infection per six months, seven clusters grew from a pair to a cluster with up to seven infections. The largest CRF01_AE cluster showed an average growth rate of 0.55 infections per six‐month interval. In contrast, six subtype B clusters grew at a rate of >1.5 infections per six‐month interval, including the largest cluster which consisted of an increase of 22 infections from seven to 29 in five years. Seventy‐eight clusters grew from being a pair to a cluster with up to nine infections.

Notably, numerous clusters did not grow continuously over time but rather one or two infections were added after prolonged time intervals. For example, one subtype B cluster consisting of 14 infections contained 13 in June 2013 and one in 2018. One CRF01_AE cluster contained three infections in December 2013 and expanded to four by June 2016 and five by June 2018 (Figure 3B). In this cohort, it was not uncommon for HIV‐1 infections to be diagnosed one year or more after transmission, as reflected by the stage of infection at diagnosis; 44% of subtype B and 60% of CRF01_AE infections were in individuals in the non‐early stages of their infection (Table 1). Within subtype B clusters, 50 sequences were obtained from individuals during the non‐early infectious stages that had a sampling time gap of >1 year to the next most recent sequence in the same cluster. In addition, we identified late additions in 47 clusters (45% of the 104 growing clusters) that were linked to other infections from individuals in their early stage of infection. Also, 42% of the 45 clusters (n = 19) contained a time gap of 2.5 to 4.5 years and 16% of clusters (n = 7) contained a time gap of five to six years despite infections being derived individuals in the early stage of infection. For CRF01_AE the largest time interval between sampling in the 11 growing clusters was three years. This was found in two clusters (18%). In both instances, the infections were among individuals in their non‐early stages of infection. In contrast, 36% of growing CRF01_AE clusters (n = 4) contained a time gap of 1.5 to 2.5 years that was associated with infections among individuals during their early stage of infection.

### 3.4 Infections from the early stage are significantly associated with growing clusters

Demographic differences were investigated within potentially extinct clusters and growing clusters for both subtypes (Table S1). For this analysis, 11 subtype B and two CRF01_AE clusters which appeared to be growing in the time period 2013 to 2018 but which contained exclusively infections among individuals in their advanced stage infection were redefined as potentially extinct (Figure 3B). This resulted in 93 growing clusters and 11 potentially extinct clusters for subtype B, and nine growing and nine potentially extinct clusters for CRF01_AE. A significant positive correlation was found for subtype B infections from the advanced stage being associated with potentially extinct clusters (*p* < 0.05, Figure S3), however, this might be biased due to the definition of clusters containing sequences sampled after 2013 from individuals during advanced infections being potentially extinct. Interestingly, no difference was found for infections among Australian‐born and non‐Australian‐born individuals being more likely associated with growing clusters for either subtype. However, subtype B infections among individuals born in “other” region were more likely to be found in extinct clusters (*p* < 0.05). In contrast, CRF01_AE infections reported to have been acquired outside Australia were associated with extinct clusters (*p* < 0.05), as well as infections acquired via non‐MSM transmission (*p* < 0.01).

## 4 Discussion

We used annotated HIV‐1 genomic sequence data and performed a phylogenetic analysis to describe cluster growth over time in NSW, Australia, and how transmission dynamics are influenced more by individuals’ demographics than virus subtype. Specifically, we show how infections forming clusters are more likely to have been acquired in Australia, as they are among individuals born in Australia, and those who were diagnosed at the early stage of their infection, regardless of the viral subtype. We also show that for both subtypes B and CRF01_AE, infections acquired outside Australia and among Asian‐born individuals are more likely to be singletons, and thus did not lead to ongoing local transmission.

Overall, demographics differed substantially between subtype B and CRF01_AE infections. This is an expected result due to their evolutionary history in Australia. Subtype B has always been the dominant subtype and its transmission has been endemic for over 30 years since the beginning of the HIV‐1 pandemic in the 1980s [1]. Thus, it is not surprising that we found that the majority of subtype B infections that were reported to have been acquired within Australia were in Australian‐born individuals, and were characterized by numerous local transmission clusters, all of which is expected for an established endemic disease.

In contrast, CRF01_AE, similar to other non‐B subtypes, is historically associated with heterosexual contact and acquisition outside Australia [28]. In our study, CRF01_AE was characterized by numerous sequence pairs and a majority of infections being singletons, which is more indicative of multiple introductions with only limited ongoing transmission [14]. This finding was supported by the associated demographic data, with a majority of these infections reported to be acquired outside Australia, and among individuals born in Asia. Also, we found a negative association with CRF01_AE infections in individuals reporting heterosexual transmission and being in clusters. Of the 22 CRF01_AE clusters seven (32%) contained exclusively infections among individuals reporting a transmission risk other than MSM, with the largest of these clusters only containing four infections. Our finding is similar to that of a recent study from the state of Victoria, Australia, which found that heterosexual transmissions rarely lead to more than one new infection within that state [29]. Overall, we found nine active growing clusters for CRF01_AE, of which seven contained infections from notifications in 2018. Thus, these active transmission clusters should be monitored to ensure contact tracing was successful in stopping further transmission. One of these clusters contained only infections among individuals reporting heterosexual transmission, and what could be evidence for one single active growing heterosexual transmission cluster in NSW. The remaining six clusters consisted mainly of infections among individuals reporting MSM transmission and we found here that MSM transmission was positively associated with growing clusters (*p* < 0.01) for CRF01_AE infections. An older study from Victoria also reported that MSM transmission was be the main risk factor for transmission clusters. That study also found an increase in MSM transmission for non‐B subtype infections [30].

This dominance of MSM transmission was more evident for subtype B infections. Of the 157 B clusters found here, 87 (55%) contained exclusively infections among individuals reporting MSM transmission with a maximum of 17 sequences. Five of these MSM‐only clusters also had a faster than average growth rate (>1.5 sequences per 6‐months). The identification of such fast‐growing clusters is crucial for epidemic control and should be prioritized for public health interventions [31]. Only two subtype B clusters were found that did not contain any infections attributed to MSM transmission, but both of these were classified as extinct.

We show here a strong positive association for infections from early stage being in clusters for both subtypes. Thus, a large proportion of HIV‐1 transmission occurs shortly after HIV‐1 acquisition. This short window is characterized by an asymptomatic phase and usually higher viraemia that facilitates transmission [32]. Notably, timely HIV‐1 diagnoses and immediate access to therapy has been successfully advocated in NSW for prevention of HIV‐1 transmission [33]. A NSW study that analysed the performance of a rapid HIV test among men found that among 94 MSM diagnosed in 2013 to 2014, 39% were diagnosed within three months of infection [34]. NSW data reporting on follow‐up at six months after diagnosis show that among people diagnosed between January and June 2019 the median time to treatment was only 16 days after diagnosis [3]. Nevertheless, we observed here both the presence “late diagnoses,” that is sequences sampled during the advanced stage of infection being linked to clusters, and “late additions,” that is sequences sampled during the early stage of infection but >1 year after the previous most closely related sequence.

We identified 11 subtype B and two CRF01_AE clusters that by molecular data alone were seemingly growing between 2013 and 2018, but these additional sequences were from infections that likely occurred over seven years prior suggesting these clusters are in fact extinct. Importantly, notifications from people who are diagnosed with HIV‐1 more than one year after a transmission event pose challenges for timely monitoring of cluster growth, as these infections occurred some time prior to diagnosis. Such late diagnoses are further problematic for public health interventions as this delays access to treatment, and control measures such as contact tracing are less likely to be effective, as contact tracing is most successful when conducted soon after a transmission event [35]. In addition, we identified 47 clusters that contained a total of 116 infections exclusively among individuals in their early stage of infection but had a large time gap (>1 year) to the most recent previous sequence in the same cluster. Thirteen percent of these sequences had a time gap of >4 years. Such a delay in HIV‐1 notifications could have different reasons, most probable being missing data. First, it is probable that these sequences represent notifications from new infections passed on from undiagnosed individuals. We know that approximately 11% of people living with HIV‐1 living in Australia are undiagnosed and these undiagnosed infections are one of the major contributors of new infections [36]. Second, we miss sequences from inter‐state transmission that could “fill‐in” the time gaps observed. A study by Castley et al. reported the presence of inter‐state transmission with approximately 40% of identified clusters containing at least two infections from different states [37]. Sequence data from that study were included here but lacked associated demographic data and only included sequences up to 2012, thus, it did not cover our period of cluster growth analysis. Finally, we cannot exclude significant uncertainties for the estimates of time since seroconversion, which could also explain some of the large time gaps observed. Using CD4 data alone might not be sufficient to estimate time point of infection. Studies of rates of CD4 decline have shown that various factors can influence the speed of decline. For example co‐infection with HCV can increase the rate of decline of CD4 cells, [38] and CD4 counts may vary between different geographic areas with faster CD4 decline observed in Asia compared to Europe [39].

Our study has some limitations: First, our cluster analysis was rather conservative by implementing a 100% NSW‐specific node cut off and only comparing “phylogenetically” stable clusters, whereas other studies have used a cut‐off of 80% [5]. However, we believe that a cut off of 100% is more suitable and precise for the robust identification of sequence pairs and small clusters (<10 sequences). Second, the data set used lacks sequence data from other Australian states, thus, we may have missed potential transmissions from interstate. Also, we show here that for both subtypes B and CRF01_AE, only infections among individuals diagnosed during the early stage of infection were associated with clusters, whereas infections among individuals diagnosed during the advanced stage of infection were associated with being singletons. However, this could be due to a bias in the data as contact tracing and hence “linked infections” is more successful in the first months after transmission, that is during early stages of infection. Also infections notified early are more likely to have been acquired locally via local transmission and having a greater potential for matching in the data set. Thus, we might be simply missing sequences linking to infections from advanced stages in our data. Finally, we lack demographic data regarding the ethnicity and travel history of people living with HIV, which could have helped assess the impact of migration and travel on HIV infection rates and transmission.

## 5 Conclusions

We report on subtype‐specific transmission dynamics with subtype B being dominated by larger clusters and CRF01_AE by sequence pairs. Infections among these two subtypes also differ in their associations with different demographic factors, particularly the transmission risk factor and stage of infection at diagnosis. Importantly, for both subtypes, we identified numerous active clusters that could be the focus of increased monitoring by public health to avoid an expansion of new HIV‐1 infections within the state. We show here the importance of utilizing molecular data for prevention of transmission and to inform the HIV‐1 public health response.

## Competing Interests

FJL has received educational grants and sponsorship from ViiV Healthcare, Gilead Sciences and MSD, speaker fees from AstraZeneca, MSD and the Australasian Society for HIV, Viral Hepatitis and Sexual Health Medicine (ASHM), and provided consultancy and served on advisory boards for Viiv Healthcare, MSD and Calimmune Australia. All other authors report no conflict of interest.

## Authors’ Contributions

Conceptualization: FDG, ANP, PK, NF, KP, JH, BBB and ADK. Data curation: FDG, ANP, AS and ADK. Sequence data: AC, HS, FJL, DED. Demographic data: CS, SJN, NF, KP and JH. Funding acquisition: FDG, AEG and ADK. Methodology: FDG, ANP, PK, JLG, Supervision: ADK. Visualization: FDG. Writing – original draft: FDG, ANP, JLG and ADK. All authors have read and approved the manuscript.

## Supporting information


**Figure S1.** Data selection. A flow chart describing the procedure for the sequence selection for subtype B (left) and CRF01_AE (right). Sequences were extracted from the NSW HIV database and split according to time point sampled forming 12 data subsets for each subtype. Global sequences were then added to each of the data subsets and a phylogenetic tree was estimated for each. Clusters were defined as nodes containing only NSW sequences (light blue) within the global phylogeny (none NSW sequences are shown in grey). Sequences pairs were defined as nodes containing two NSW sequences (yellow), all other NSW sequences were defined to be singletons (green). Example of cluster growth over time. Branches in light blue represent a cluster with six sequences identified in data subset Dec 2015, and which grew by two sequences in the subsequent data subset. Branch lengths represents nucleotide substitutions per site.
**Figure S2.** Phylogeny. Maximum likelihood trees harbouring all sequence data used in this study, i.e. data subset Dec 18, are shown for subtype B (left) and CRF01_AE (right). Grey global sequences, black singleton sequences, yellow, sequence pairs, and light blue clusters. Branch lengths indicates nucleotide substitutions per site.
**Figure S3.** Correlations between sequence demographics and cluster association for subtype B. Plots shows correlations for each cell (demographic vs growing cluster, potentially extinct cluster, sequence pair). As there were less than five sequences with the PWID transmission risk factor, this risk factor category was combined with “Other”. Positive values (blue) depict a positive association, negative values (red) depict a negative association. The bigger the square, the stronger the association. Region acquired contained a large proportion of missing data points (Table S1), which were excluded here. P values represent the overall statistic for the corresponding category. MSM, men who have sex with men, Heterosexual; PWID, person who inject drugs.
**Table S1.** Demographic factors for sequences associated with growing clusters, potentially extinct clusters, or pairs

## Data Availability

Due to the high sampling density in this study only a random subset of 10% of sequence data has been made available via National Center for Biotechnology Information (NCBI) under the accession numbers MW167298 ‐ MW 167644. This will avoid potential identification of complete transmission networks and ensures individuals data privacy.
