# Supplementary material for: Subtype‐specific differences in transmission cluster dynamics of HIV‐1 B and CRF01_AE in New South Wales, Australia
Source: J Int AIDS Soc. 2021 Jan 20;24(1):e25655. doi: 10.1002/jia2.25655 (PMC7817915; doi:10.1002/jia2.25655)
Supplement: Supplementary file 1 — Figure S1. Data selection. A flow chart describing the procedure for the sequence selection for subtype B (left) and CRF01_AE (right). Sequences were extracted from the NSW HIV database and split according to time point sampled forming 12 data subsets for each subtype. Global sequences were then added to each of the data subsets and a phylogenetic tree was estimated for each. Clusters were defined as nodes containing only NSW sequences (light blue) within the global phylogeny (none NSW sequences are shown in grey). Sequences pairs were defined as nodes containing two NSW sequences (yellow), all other NSW sequences were defined to be singletons (green). Example of cluster growth over time. Branches in light blue represent a cluster with six sequences identified in data subset Dec 2015, and which grew by two sequences in the subsequent data subset. Branch lengths represents nucleotide substitutions per site. Figure S2. Phylogeny. Maximum likelihood trees harbouring all sequence data used in this study, i.e. data subset Dec 18, are shown for subtype B (left) and CRF01_AE (right). Grey global sequences, black singleton sequences, yellow, sequence pairs, and light blue clusters. Branch lengths indicates nucleotide substitutions per site. Figure S3. Correlations between sequence demographics and cluster association for subtype B. Plots shows correlations for each cell (demographic vs growing cluster, potentially extinct cluster, sequence pair). As there were less than five sequences with the PWID transmission risk factor, this risk factor category was combined with “Other”. Positive values (blue) depict a positive association, negative values (red) depict a negative association. The bigger the square, the stronger the association. Region acquired contained a large proportion of missing data points (Table S1), which were excluded here. P values represent the overall statistic for the corresponding category. MSM, men who have sex with men, Heterosexual; PWID, person who inj [file JIA2-24-e25655-s001.docx]

**Supplementary figure S1.** Data selection. A flow chart describing the procedure for the sequence selection for subtype B (left) and CRF01_AE (right). Sequences were extracted from the NSW HIV database and split according to time point sampled forming 12 data subsets for each subtype. Global sequences were then added to each of the data subsets and a phylogenetic tree was estimated for each. Clusters were defined as nodes containing only NSW sequences (light blue) within the global phylogeny (none NSW sequences are shown in grey). Sequences pairs were defined as nodes containing two NSW sequences (yellow), all other NSW sequences were defined to be singletons (green). Example of cluster growth over time. Branches in light blue represent a cluster with six sequences identified in data subset Dec 2015, and which grew by two sequences in the subsequent data subset. Branch lengths represents nucleotide substitutions per site.

**Supplementary figure S2.** Phylogeny. Maximum likelihood trees harbouring all sequence data used in this study, i.e. data subset Dec 18, are shown for subtype B (left) and CRF01_AE (right). Grey global sequences, black singleton sequences, yellow, sequence pairs, and light blue clusters. Branch lengths indicates nucleotide substitutions per site.

**Supplementary figure S3.** Correlations between sequence demographics and cluster association for subtype B. Plots shows correlations for each cell (demographic vs growing cluster, potentially extinct cluster, sequence pair). As there were less than five sequences with the PWID transmission risk factor, this risk factor category was combined with “Other”. Positive values (blue) depict a positive association, negative values (red) depict a negative association. The bigger the square, the stronger the association. Region acquired contained a large proportion of missing data points (supplementary table S1), which were excluded here. P values represent the overall statistic for the corresponding category. MSM = men who have sex with men, Heterosexual, PWID = person who inject drugs.

Supplementary Table S1. Demographic factors for sequences associated with growing clusters, potentially extinct clusters, or pairs. As there were less than five sequences with the PWID transmission risk factor, this risk factor category was combined with “Other”. For CRF01_AE region born data from individuals born in Europe is combined with others and for stages of infection CD4 <500, CD4 <350, and advanced data is combined due to low sample size.

|  | Subtype B | |  | CRF01_AE | |
| --- | --- | --- | --- | --- | --- |
|  | Growing (n=566) | Extinct (n=167) |  | Growing (n=48) | Extinct (n=28) |
| *Sex* | | | | | |
| Male | 0.80 (454) | 0.92 (155) |  | 0.88 (42) | 0.82 (23) |
| Female | 0.02 (11) | 0.03 (5) |  | <0.1 (<5) | <0.2 (<5) |
| No data | 0.18 (101) | 0.04 (7) |  | <0.1 (<5) | <0.2 (<5) |
| *Region acquired* |  |  |  |  |  |
| Australia | 0.77 (436) | 0.53 (89) |  | 0.77 (37) | 0.46 (13) |
| Not Australia | 0.06 (33) | 0.05 (8) |  | 0.15 (7) | 0.39 (11) |
| No data | 0.17 (97) | 0.42 (70) |  | <0.1 (<5) | <0.2 (<5) |
| *Region born* |  |  |  |  |  |
| Australian | 0.67 (380) | 0.69 (115) |  | 0.42 (20) | 0.32 (9) |
| Non-Australian | 0.29 (162) | 0.24 (39) |  | 0.56 (27) | 0.68 (19) |
| Asia | 0.1 (56) | 0.05 (9) |  | 0.42 (20) | 0.46 (13) |
| Europe | 0.09 (53) | 0.05 (9) |  | - | - |
| Other | 0.09 (53) | 0.13 (21) |  | 0.15 (7)* | 0.21 (6)* |
| No data | 0.04 (24) | 0.08 (13) |  | <0.1 (<5) | <0.2 (<5) |
| *Transmission risk factor* | |  |  |  |  |
| MSM | 0.89 (502) | 0.84 (140) |  | 0.85 (41) | 0.54 (15) |
| Heterosexual | 0.05 (31) | 0.08 (14) |  | <0.1 (<5) | 0.25 (7) |
| PWID/Other | 0.06 (33) | 0.08 (13) |  | <0.1 (<5) | 0.21 (6) |
| *Stage of infection at diagnosis* | |  |  |  |  |
| Early | 0.65 (367) | 0.60 (100) |  | 0.65 (31) | 0.43 (12) |
| CD4 <500 | 0.11 (64) | 0.07 (11) |  | - | - |
| CD4 <350 | 0.10 (54) | 0.08 (13) |  | - | - |
| Advanced | 0.12 (70) | 0.20 (33) |  | 0.33 (16)* | 0.54 (15)* |
| No data | 0.02 (11) | 0.06 (10) |  | <0.1 (<5) | <0.2 (<5) |

MSM men who have sex with men

PWID person who inject drugs
